# Supplementary material for: Reconciling Mining with the Conservation of Cave Biodiversity: A Quantitative Baseline to Help Establish Conservation Priorities
Source: PLoS One. 2016 Dec 20;11(12):e0168348. doi: 10.1371/journal.pone.0168348 (PMC5173368; doi:10.1371/journal.pone.0168348)
Supplement: S1 Dataset — (ZIP) [file pone.0168348.s002.zip › Taxa/Serra Norte/SN_2013/PES/Lista PESE-03.pdf]

CAVIDADE PESE-0003

Classe	Ordem	Fam/Outros	Gên/Outros	Espécie	S-U	U-S	Zon.
Arachnida	Acari	Bdellidae		sp.1	X		E
Arachnida	Acari	Rhagidiidae		sp.1		X	E
Arachnida	Amblypygi	Phrynidae	*Heterophrynus*	*longicornis*		1	E
Arachnida	Araneae	Pholcidae	*Mesabolivar*	*eberhard*	X	X	E
Arachnida	Opiliones	Sclerosomatidae	*Prionostema*	sp.1	X	X	E
Entognatha	Collembola	Paronellidae		sp.1		X	E
Insecta	Coleoptera	Coccinellidae		sp.1	X		E
Insecta	Coleoptera	Scydmaenidae		sp.1	X		E
Insecta	Diptera	Culicidae		jovem	X		E
Insecta	Diptera	Drosophilidae	*Drosophila*	*eleonorae*		X	E
Insecta	Diptera	Psychodidae	*Pericoma*	sp.	X	X	E
Insecta	Diptera	Psychodidae	*Telmatoscopus*	sp.	X	X	E
Insecta	Diptera	Tipulidae	Tipulinae	sp.	X		E
Insecta	Heteroptera	Veliidae	*Rhagovelia*	jovem	X		E
Insecta	Hymenoptera	Formicidae	*Camponotus*	sp.1		X	E
Insecta	Hymenoptera	Formicidae	*Nylanderia*	sp.1	X		E
Insecta	Hymenoptera	Formicidae	*Nylanderia*	sp.3		X	E
Insecta	Hymenoptera	Formicidae	*Pheidole*	sp.2		X	E
Insecta	Isoptera	Termitidae	*Nasutitermes*	sp.	X		E
Insecta	Lepidoptera	Noctuoidea		sp.2	X		E
Insecta	Psocoptera	Psyllipsocidae	*Psyllipsocus*	sp.1		X	E
Mammalia	Chiroptera	Phyllostomidae	*Carollia*	*perspicillata*		17	E
Mammalia	Chiroptera	Phyllostomidae	*Phyllostomus*	sp.		1	E
Reptilia	Squamata	Gymnophthalmidae	aff. *Neusticurus*	sp.		1	E
